# Computer-vision analysis of craniofacial dysmorphology in 22q11.2 deletion syndrome and psychosis spectrum disorders

**DOI:** 10.1186/s11689-024-09547-8

**Published:** 2024-06-25

**Authors:** David R. Roalf, Donna M. McDonald-McGinn, Joelle Jee, Mckenna Krall, T. Blaine Crowley, Paul J. Moberg, Christian Kohler, Monica E. Calkins, Andrew J.D. Crow, Nicole Fleischer, R. Sean Gallagher, Virgilio Gonzenbach, Kelly Clark, Ruben C. Gur, Emily McClellan, Daniel E. McGinn, Arianna Mordy, Kosha Ruparel, Bruce I. Turetsky, Russell T. Shinohara, Lauren White, Elaine Zackai, Raquel E. Gur

**Affiliations:** 1 Brain Behavior Laboratory, Department of Psychiatry, Perelman School of Medicine, University of Pennsylvania, Philadelphia, Pennsylvania USA; 2 Lifespan Brain Institute, Department of Child and Adolescent Psychiatry and Behavioral Sciences, Children’s Hospital of Philadelphia, Philadelphia, USA; 3 22q and You Center at the Children’s Hospital of Philadelphia, Philadelphia, USA; 4 FDNA, Boston, MA USA; 5 Penn Statistics in Imaging and Visualization Endeavor (PennSIVE), Perelman School of Medicine, University of Pennsylvania, Philadelphia, PA USA; 6 Center for Biomedical Image Computing & Analytics (CBICA), Perelman School of Medicine, University of Pennsylvania, Philadelphia, PA USA; 7 Neuropsychiatry Section, Department of Psychiatry, 5th Floor, Richards Building, 3700 Hamilton Walk, Philadelphia, PA 19104 USA

**Keywords:** Psychosis, Minor physical anomalies, 22q11.2 deletion syndrome, Face, Computer-vision, Schizophrenia, Clinical high-risk psychosis

## Abstract

**Background:**

Minor physical anomalies (MPAs) are congenital morphological abnormalities linked to disruptions of fetal development. MPAs are common in 22q11.2 deletion syndrome (22q11DS) and psychosis spectrum disorders (PS) and likely represent a disruption of early embryologic development that may help identify overlapping mechanisms linked to psychosis in these disorders.

**Methods:**

Here, 2D digital photographs were collected from 22q11DS (*n* = 150), PS (*n* = 55), and typically developing (TD; *n* = 93) individuals. Photographs were analyzed using two computer-vision techniques: (1) DeepGestalt algorithm (Face2Gene (F2G)) technology to identify the presence of genetically mediated facial disorders, and (2) Emotrics—a semi-automated machine learning technique that localizes and measures facial features.

**Results:**

F2G reliably identified patients with 22q11DS; faces of PS patients were matched to several genetic conditions including FragileX and 22q11DS. PCA-derived factor loadings of all F2G scores indicated unique and overlapping facial patterns that were related to both 22q11DS and PS. Regional facial measurements of the eyes and nose were smaller in 22q11DS as compared to TD, while PS showed intermediate measurements.

**Conclusions:**

The extent to which craniofacial dysmorphology 22q11DS and PS overlapping and evident before the impairment or distress of sub-psychotic symptoms may allow us to identify at-risk youths more reliably and at an earlier stage of development.

**Supplementary Information:**

The online version contains supplementary material available at 10.1186/s11689-024-09547-8.

## Background

The 22q11.2 deletion syndrome (22q11DS) is the most common chromosomal microdeletion in humans [1]. 22q11DS is associated with an increased risk for psychiatric disorders [2–6], including psychosis [7, 8] with similar symptoms to individuals with idiopathic schizophrenia (SZ) [3]. Indeed, about 1–2% of cases of idiopathic SZ have 22q11.2 deletions [9]. Thus, targeted approaches detailing specific dysfunction in 22q11DS may elucidate critical mechanisms relevant to psychosis. Specifically, capturing abnormalities common to individuals at-risk for psychosis and with a genetic risk to psychosis, such as 22q11DS, may elucidate risk and resilience for psychosis.

Minor physical anomalies (MPAs) are congenital morphological abnormalities associated with disruptions of fetal development [10] and are common in 22q11DS and psychosis. MPAs encompass deviations of somatic features including the eyes, ears, mouth and head [11]. Abnormalities of facial morphology likely represent a disruption of early embryologic development, making identification of MPAs a promising entry point for understanding neurodevelopmental abnormalities associated with 22q11DS and psychosis. Given that there are common developmental disturbances in both 22q11DS and psychosis, a higher prevalence and/or distinct patterns of MPAs may be present in 22q11DS patients with psychosis symptoms.

Knowledge regarding the embryogenesis of the face not only allows insight into normal variations in facial structure, but also provides an understanding of how and when congenital anomalies occur [12, 13]. This craniofacial embryogenesis can be considered a program of complex, well-orchestrated changes in cranial and facial morphology and a cascade of biochemical events that control the anatomic changes. Early in fetal life, the face and forebrain evolve in “embryological intimacy” [13, 14]. This developmental unity is thought to undergird both neurodevelopmental disorders and disorders involving facial dysmorphogenesis and may be associated with clinical and cognitive deficits in adulthood among these developmental conditions [15]. Disorders falling in this classification range from major chromosomal abnormalities, such as Down syndrome, to less readily recognizable conditions, such as 22q11DS. Consistent with this intertwining of neurodevelopment and craniofacial morphogenesis, individuals with facial dysmorphogenesis such as cleft lip/palate have abnormalities of brain structure [16] and display cognitive deficits [17].

Recently, the application of anthropometric [18–21] and 3D techniques [22–25] has revealed subtle facial dysmorphology in SZ. Previous investigations reported higher frequency of malformations of the limbs, face, and eyes in adults with SZ [26, 27]. These initial correlations were followed by several studies reporting higher incidence of malformations of the head, eyes, ears, mouth, hands, and feet in SZ compared to typically developing (TD) individuals [28, 29]. More recent evidence indicates that the presence of abnormalities of the face is associated with a two-fold increase in the risk for psychosis [30, 31]. Overall, results of the aforementioned studies converge in suggesting that the topography of craniofacial dysmorphology may reflect subtle disruption of a critical trajectory of embryonic-fetal craniofacial growth, likely during organogenesis, and particularly along the midline, which may be relevant for elucidating risk for significant psychopathology.

In the current investigation, we use deep learning and computer-vision analysis of facial morphology to compare developmental facial features in individuals with 22q11DS—with and without psychopathology—to individuals with idiopathic psychosis spectrum disorder (PS) and TD. Using two-dimensional (2D) facial photographs, we hypothesized that computer-vision analysis will reliably detect individuals with 22q11DS, that the detection of dysmorphic features associated with 22q11DS will be more prominent in individuals with idiopathic PS than TD, that specific anatomical features along the facial midline will be dysmorphic in both 22q11DS and PS as compared to TD and that when combined computer-vision approaches help distinguish individuals with psychosis from TD.

## Methods

### Participants

The sample included 150 (41% female) individuals with 22q11DS, mean age 17 (±10) years, and 148 non-deleted individuals: 55 (42% female) PS, mean age 28 (±9), and 93 (32% female) TD, mean age 34 (±11; See Table 1). Participants were recruited at two collaborating sites: the 22q and You Center at the Children’s Hospital of Philadelphia (CHOP) and the Lifespan Brain Institute and Brain Behavior Laboratory at Penn Medicine. All those with 22q11DS participants had a confirmed chromosome 22q11.2 deletion using fluorescence in situ hybridization (FISH), comparative genomic hybridization, multiplex ligation-dependent probe amplification, or Single Nucleotide Polymorphism microarray [32]. PS and TD were not tested for 22q11DS as the prevalence of 22q11DS in idiopathic PS is low (~ 1%) [33]. General enrollment criteria included: expressing interest in providing a photo, proficiency in English, ambulatory and in stable health, and physical capability of having a photograph taken. Participants provided informed consent/assent and the study procedures were approved by the Institutional Review Boards at CHOP and Penn. Additional participant characteristics are detailed in Table 1.

**Table 1:** Participant characteristics

Group	N	Age range	Age	SZ/CR	F/M	Black/White/Other	GAF	MMSE	A-D Deletion
22q11DS	150	1–45	17 (10)	NA	62/88	14/129/7	63 (16)	22 (6)	96%
PS	55	15–50	28 (9)	38/17	23/32	29/20/6	59 (17)	27 (5)	NA
TD	93	18–72	34 (11)	NA	30/63	37/54/2	85 (7)	29 (2)	NA

Out of 150 22q11DS subjects, 6 were missing age data, 22 were missing GAF data, 39 were missing MMSE data. Out of 55 PS subjects, 26 were missing GAF data and 14 were missing MMSE data. Out of 93 TD subjects, 1 was missing age data, 70 were missing GAF, and 52 were missing MMSE. Abbreviations: 22q11DS = 22q11.2 deletion syndrome; PS = psychosis spectrum; TD = typically developing; SZ = schizophrenia; CR = clinical high risk for psychosis; F = female; m = male; GAF = global assessment of function; MMSE = Mini-Mental Status Examination; A-D Deletion = presence of deletion from the A-D section on the long arm (q) of chromosome 22q; NA = not available

### Clinical Assessment

Clinical phenotyping was completed using structured clinical interviews and standardized clinical questionnaires. Threshold psychotic disorders were determined using DSM-IV-TR criteria [34], and subthreshold psychotic symptoms were determined using the Scale of Prodromal Symptoms [35–37]. In the PS group, 38 individuals met threshold criteria for psychosis (e.g., schizophrenia) and the remaining 17 met subthreshold criteria. Participants with 22q11DS were evaluated for psychosis when referred by a clinician at the 22q and You Center. Seventy-seven (51%) individuals with 22q11DS exhibited PS symptoms (22q11DS+); 71 (48%) exhibited no PS symptoms (22q11DS-) and two were not assessed for psychopathology.

Clinical assessments were administered by interviewers trained by a clinical psychology faculty member. Narrative assessment summaries were presented at consensus case conferences where diagnoses were finalized by consensus of two or more doctoral level clinicians. Measures of global functioning (Global Assessment of Functioning; GAF [38] and general cognitive ability (Mini-Mental Examination; MMSE [39] were collected when possible.

### Two-dimensional (2D) photographs

**Fig. 1:**
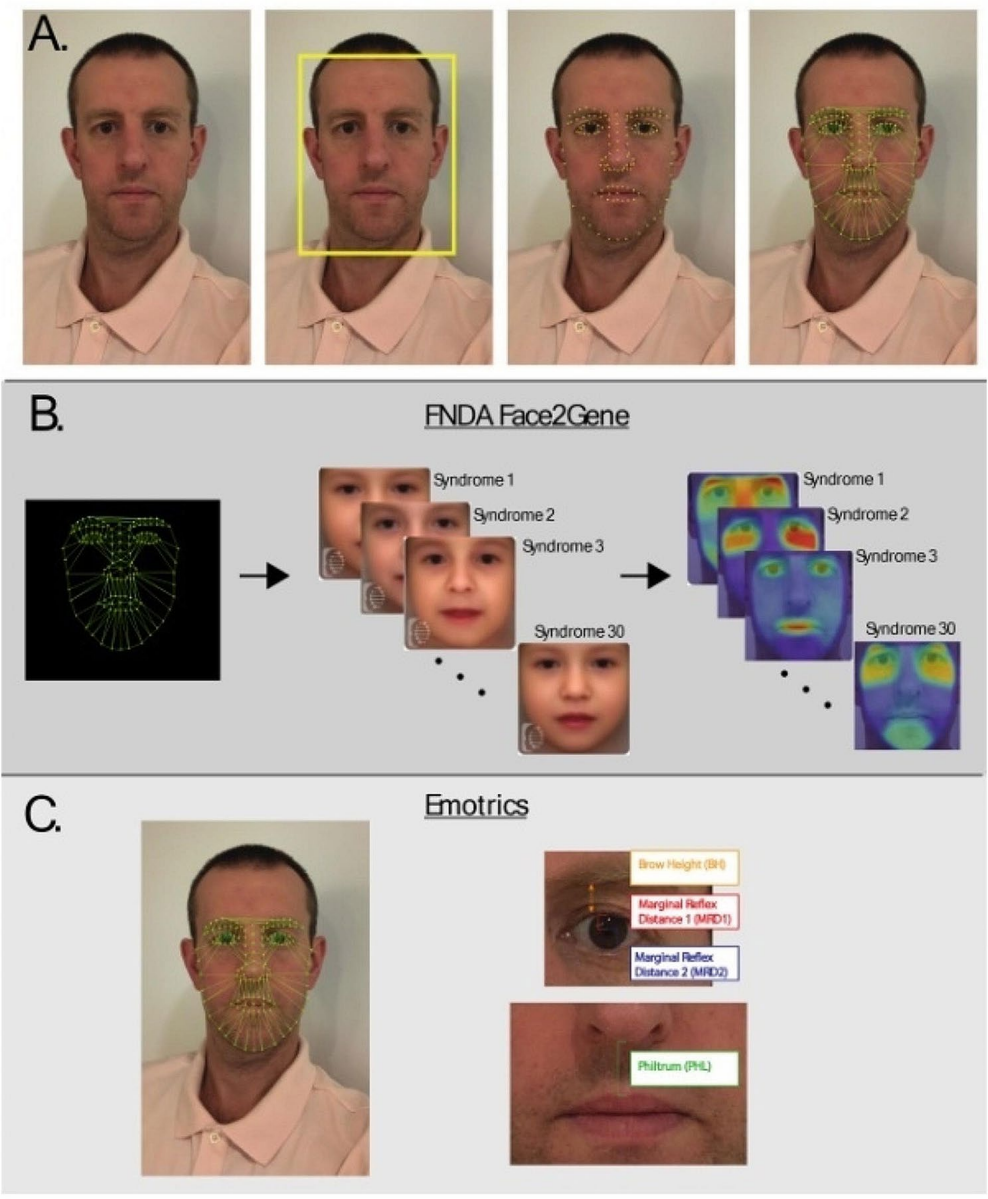
Front facing 2D photographs (**A**) were collected using digital photography. FDNA Face2Gene (**B**) and Emotrics (**C**) were used to identify facial features. Face2Gene enables detection of dysmorphic features and recognizable patterns of human malformations while Emotrics localizes facial landmarks and computation regional measures of facial features. *Note: The image used in this Figure is that of Dr. David Roalf and is used only to illustrate the methods used. Dr. Roalf provides permission for the use of his face in this image*

Front-facing 2D pictures were taken of individuals using standard digital photography (Fig. 1A). Participants were instructed to maintain a neutral expression while sitting or standing in front of a neutral background. In most cases, 2D photographs were taken by study staff during clinical or research visits. Fifteen photos from persons with 22q11DS were not collected by study staff but were provided by the participant or a family member. There were no differences in facial measurement metrics between these two types of photos. All photographs were visually inspected for quality and cropped to include each individual’s head and face only. Facial images were verified to be detectable by an automatic facial recognition algorithm (Emotrics Software). Any photo failing QA was discarded.

### Deep learning and computer-vision analysis of facial morphology

*Using the DeepGestalt algorithm in the Face2Gene (F2G) platform FDNA Inc., Boston MA, USA); (*https://www.fdna.com))

FDNA has cataloged over 20,000 patients and has demonstrated strong prediction for over 300 syndromes [40]. Using the DeepGestalt technology implemented in FDNA’s F2G platform facial phenotypes were identified (Fig. 1B). DeepGestalt combines facial recognition software with clinical information (if available) to detect dysmorphic features and recognizable patterns of human malformations in 2D facial photographs. The output of DeepGestalt is an estimate (Gestalt score) of how much the uploaded photo matches a given syndrome template within its library. DeepGestalt outputs Gestalt scores for the best 30 syndrome-matches for any given photo. Computational details of DeepGestalt are published [41].

### Emotrics

Computation of local facial dysmorphology using 2D photographs was achieved using Emotrics [42]. Emotrics (Fig. 1C) is a recently developed semi-automated machine learning technique that localizes facial landmarks and employs computation analysis using big data [43–45]. Facial landmark points are automatically placed when a front-facing 2D image is uploaded to the software. Since Emotrics was created using a database of normal faces, the auto-generated landmark points were independently confirmed and edited on all 2D pictures as needed by a trained user (MK). Two users (JJ and MK) were trained to a reliability of 0.95 using ten example faces. Facial landmarks outlined the superior border of the brow, the free margin of the upper and lower eyelids, the nasal midline, the nasal base, the mucosal edge, and vermillion-cutaneous junction of the upper and lower lips, as well as the lower two-thirds of the face. The center of the eyes and borders of the iris borders were adjusted as needed to improve accuracy.

Emotrics automatically computes three literature-established set of facial measurements: bilateral eyebrow height (BH), palpebral marginal reflex distance 1 (MRD1) and 2 (MRD2) [42]. In addition, using the point-based text output of Emotrics, we computed measurement of the philtrum (PHL) using average distance between the nasal base and upper lip. Brow height, marginal reflex distance 1 and 2 were averaged across the left and right sides of the face. Thus, four features were used in the final analysis. Emotrics is a freely available, open-source software suite that can be downloaded from github (https://github.com/dguari1/Emotrics).

### Statistical analysis

Participant characteristics were compared using linear regression analysis and chi-square tests to identify differences between diagnostic groups (Table 1). Facial features were analyzed using a series of Analyses of Variance (ANOVAs), Receiver Operating Characteristics Curve (ROC), Principal Component Analysis (PCA), and follow-up t-tests.

Gestalt scores specifically for 22q11DS syndrome were extracted for each photo and ROCs were used to measure the face validity of FDNA’ F2G algorithm for detecting 22q11DS. The expectation was that FDNA Gestalt scores for 22q11DS should be high and predictive for clinical status in laboratory-verified 22q11DS patients. ROC analyses were performed to determine how well 22q11DS Gestalt scores predicted 22q11.2 status as compared to non-deleted individuals (PS/TD).

#### Principal component analysis (PCA)

Previous studies using F2G output have limited analysis to only the expected outcome, for example how many patients with laboratory confirmed 22q11.2 deletion syndrome are accurately identified by F2G. Here we a looked beyond the expected syndrome and were interested in all possible syndromes detected by F2G. In sum, 227 unique syndromes were detected by FDNA across all 22q11DS participants.

Given the large number of syndromes detected by F2G the goal of PCA was to (1) reduce the number of variables subject to analysis while capturing most of the variance in the original set of variables; (2) capture common variance across Gestalt scores, (3) calculate principal components (PCs) and use these PCs to produce extrapolated factor scores for use in the out of sample PS and TD groups. Based on visual scree plot analysis it was determined that the first 4 principal components should be retained (Supplemental Fig. 6). Additional details on the PCA analysis are provided in the Supplemental Methods.

All PC scores and all four Emotrics measurements were used as dependent variables in analysis. Prior to analysis PC scores and Emotrics measurements were adjusted for age, sex, and race by performing a linear regression using PCs or Emotrics measures as the dependent variable and age, sex, and race as the independent variables. The adjusted residuals from these models were extracted used for group comparisons. Then, the data were analyzed using logistic regression; 95% confidence intervals were estimated using DeLong method and *n* = 2000 bootstraps. Estimated marginal means were computed using the emmeans package in R and used for follow-up contrasts. Significance levels were set to alpha = 0.05 for primary and exploratory analyses. Significance values for post-hoc comparisons of primary analyses were corrected using false-discovery rate (FDR) and are denoted in the text (p_FDR_). All statistical analyses were conducted in R version 4.0.2.

## Results

### Demographics and clinical measures

Age [F(2, 288) = 75.69, *p* < 2.2 × 10^-16^] and race distribution (X^2^(4) = 58.57, *p* = 5.79 × 10^-12^) differed by group (Table 1). As expected, patients with 22q11DS were younger and were predominantly White. TD were older than PS (*p* = 8.4 × 10^-4^). TD and PS were more racially balanced, with the PS group including a higher percentage of Black individuals (52%) as compared to TD (40%). Sex distribution did not differ by group (X^2^(2) = 2.29, *p* = 0.32). GAF scores [F(2, 177) = 23.42, *p* < 9.5 × 10^-10^] were lower in 22q11DS (*p* = 2.5 × 10^-9^) and PS (*p* = 1.3 × 10^-8^) as compared to TD but did not differ between 22q11DS and PS (*p* = 0.29). MMSE scores [F(2, 190) = 26.81, *p* < 5.5 × 10^-11^] were lower in 22q11DS as compared to PS (*p* = 3.5 × 10^-6^) and TD (*p* = 1.4 × 10^-9^), but PS and TD did not differ (*p* = 0.16).

### 22q11DS gestalt scores

**Fig. 2:**
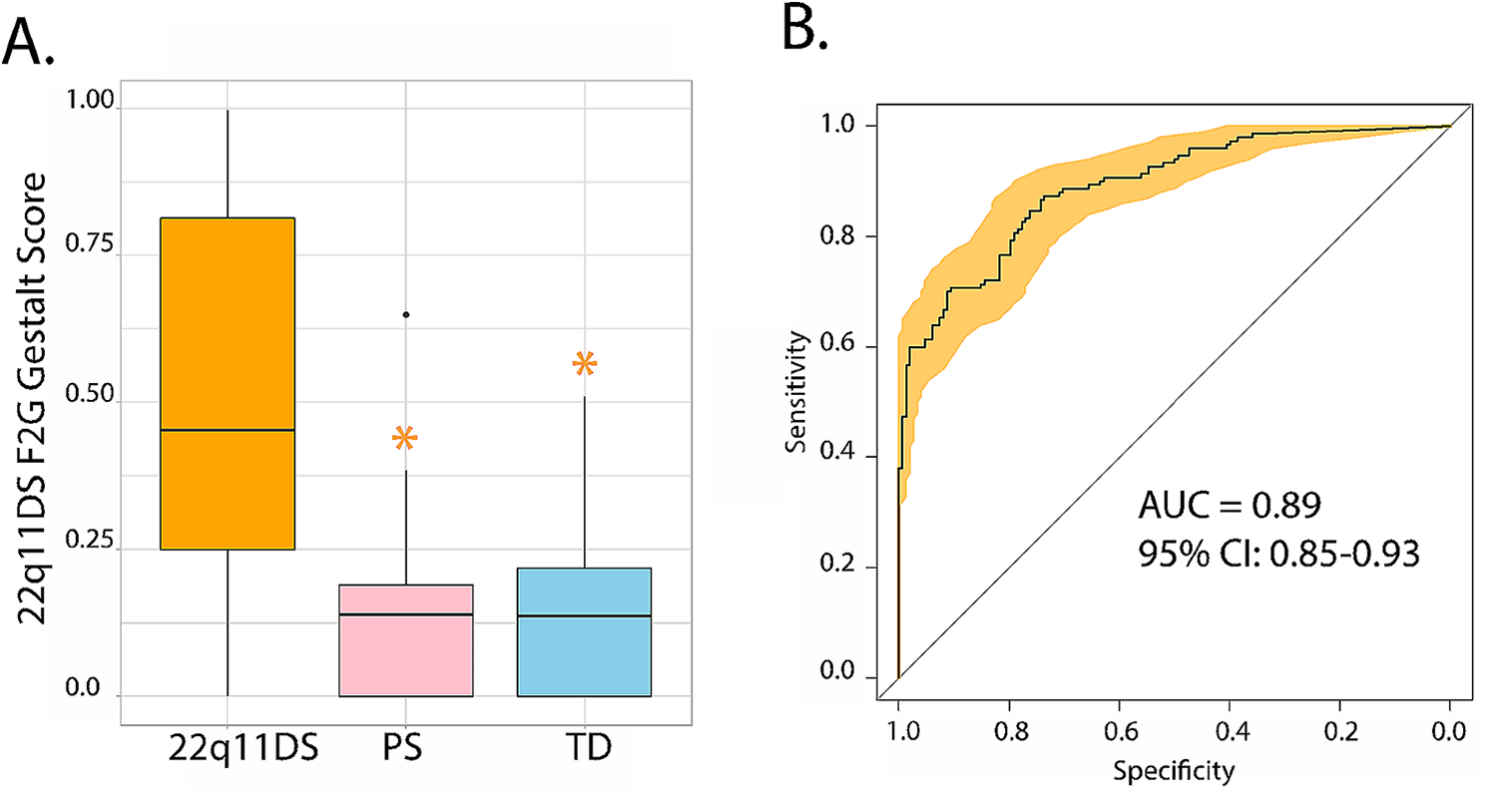
The criterion validity of F2G was high. The F2G Gestalt score was significantly higher in 22q11DS patients as compared to non-deleted PS and TD (**A**). ROC analysis of the F2G was highly accurate in differentiating deleted vs. non-deleted individuals (**B**). Outliers are black dots. *p < 0.05 as compared to 22q11DS

To test the criterion validity of F2G algorithm, we first measured its accuracy of F2G in detecting patients with a laboratory confirmed 22q11.2 deletion. F2G accurately detected 22q11DS in 99% of these individuals (Supplemental Figs. 3–5). F2G 22q11DS Gestalt scores were robust in 22q11DS (0.52±0.29) and significantly higher [F(2,284) = 67.98, *p* < 2.0 × 10^-16^] as compared to non-deleted individuals (e.g., PS or TD): PS (p_FDR_=2.0 × 10^-16^; Cohen’s d = 1.47) or TD (p_FDR_=2.0 × 10^-16^; Cohen’s d = 1.58; Fig. 2a). The 22q11DS F2G Gestalt score in PS (0.14±0.13) and TD (0.14±0.12) were low and did not differ (p_FDR_=0.97). Finally, the area-under the curve (AUC) of the ROC for identifying 22q11DS participants from non-deleted individuals using only the F2G 22q11DS Gestalt scores was high (AUC = 0.89, 95%CI: 0.85–0.93). Model sensitivity was 70%, with 91% specificity (Fig. 2b).

### PCA of F2G syndrome

PCA was performed across all participants with 22q11DS for the 30 F2G detected syndromes to determine the syndromic combinations that best represented 22q11DS participants. PCA across all syndromes identified four factors. The first factor (13.7% of the variance) had only positive loadings and was dominated by the presence of a strong 22q11.2 DS loading (Supplemental Figs. 6–8). The remaining three factors had both positive and negative loadings. The largest positive loading for the second factor was Prader-Willi Syndrome, while the largest negative was a moderate loading for Noonan Syndrome. Williams-Beuren Syndrome was the largest positive loading on the third factor, while Marfan Syndrome had the largest negative loading on the third factor. Lastly, the fourth factor was driven by a moderate positive loading for Rett Syndrome and moderate negative loading for Silver-Russell Syndrome. Loadings on each factor are shown in Supplemental Fig. 7.

**Fig. 3:**
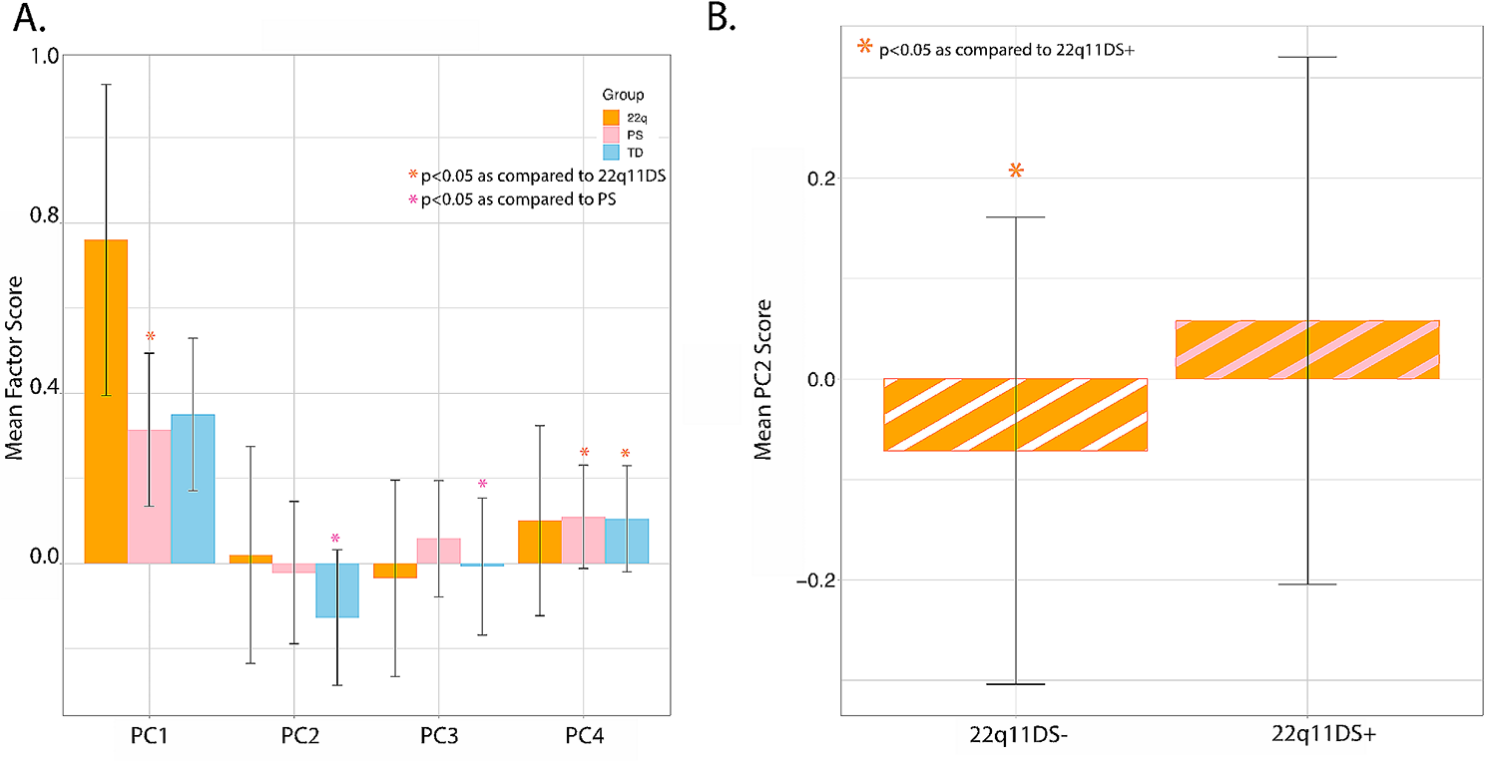
Mean (+/- SD) PC scores for F2G 22q11DS-related facial Gestalts. (**A**) 22q11DS differed from PS and TD on PC1 and PC4, while PS and TD differed on PC2 and PC3. (**B**) A comparison of 22q11DS patients with (+) and without (-) psychosis spectrum symptoms indicated PC2 scores were lower in 22q11DS+; a similar pattern was seen in non-deleted PS vs. TD (B)

Factors scores were computed for all participants using the loadings of each PC as weights in a linear combination of the relevant Gestalt scores. These PCs adjusted for age, sex, and race using linear regression creating adjusted PCs. Adjusted PCs were compared between groups (Fig. 3a). 22q11DS [F(2, 284) = 48.75, *p* < 2.2 × 10^-16^] differed from TD and PS on PC1 [22q11DS vs. TD: (p_FDR_<4.32 × 10^-12^); 22q11DS vs. PS: (p_FDR_<1.49 × 10^-10^)] and PC4 [22q11DS vs. TD: (p_FDR_<3.20 × 10^-12^); 22q11DS vs. PS: (p_FDR_<1.48 × 10^-2^)], but not on PC2 or PC3.

To determine if combinations of facial gestalts related to psychopathology within 22q11DS, PC scores were explored between 22q11DS + and 22q11DS- patients. 22q11DS + had significantly higher PC2 scores as compared to 22q11DS- [F(1, 136) = 8.20, *p* = 4.83 × 10^-3^; Fig. 3b]. No other PC scores differed between 22q11DS subgroups. Furthermore, 22q11DS + had equivalent PC2 (*p* = 0.56) and PC3 (*p* = 0.52) as non-deleted PS but differed on PC1 [F(1,125) = 41.25, *p* < 2.52 × 10^-9^] and PC4 [F(1,125) = 41.25, *p* < 3.43 × 10^-2^].

As expected, there was no difference (*p* = 0.38) in PC1 between TD and PS, as this factor is dominated by 22q11DS Gestalt score. PS had higher PC2 [F(1,141) = 7.43, *p* = 7.21 × 10^-3^] and PC3 scores [F(1,140) = 5.11, *p* = 2.53 × 10^-2^] compared to TD. PC4 scores did not differ between PS and TD (*p* = 0.98). An exploratory analysis of non-deleted PS subgroups, which included DSM diagnoses of schizophrenia (SZ; *n* = 38) and subthreshold clinical risk (CR, *n* = 17) was also performed. PC2 [F(1,124) = 9.25, *p* = 2.87 × 10^-3^] and PC3 [F(1,124) = 4.52, *p* = 3.54 × 10^-2^] scores were significantly higher in SZ as compared to TD. CR differed from TD [F(1,103) = 4.57, *p* = 3.48 × 10^-2^] and SZ on only PC4 scores [F(1,49) = 5.80, *p* = 1.98 × 10^-2^].

### Measures of the eyes and nose differ in 22q11DS, PS and TD

**Fig. 4:**
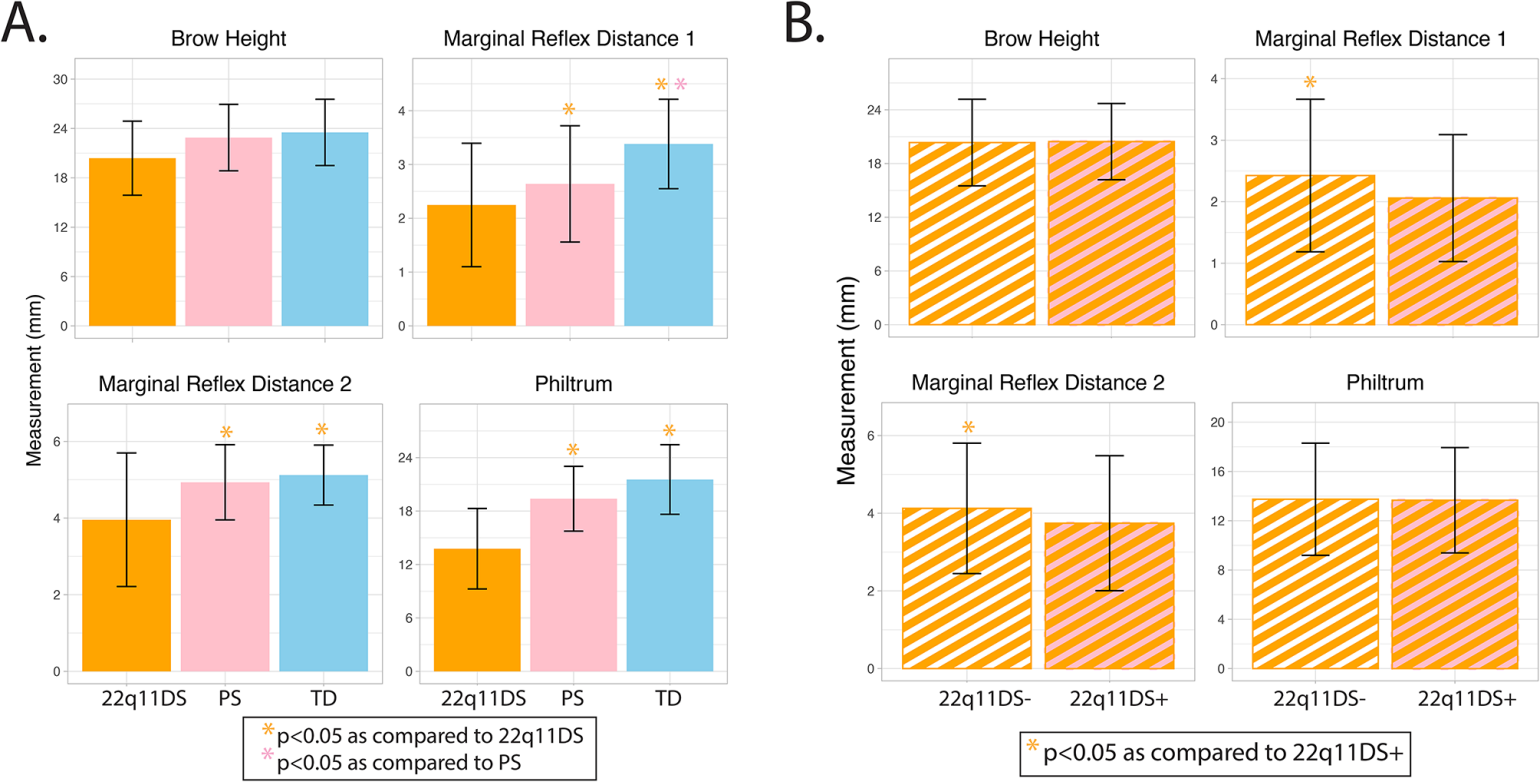
Mean (+/- SD) regional measures of the eyes and nose in 22q11DS, PS and TD. (**A**) Emotrics-derived measures of the eye (marginal reflex distance 1 and 2) and the nose (philtrum) were significantly shorter in 22q11DS as compared to PS and TD. Marginal reflex distance 1 was shorter in PS as compared to TD. (**B**) 22q11DS + had shorter marginal reflex distance 1 and 2 as compared to 22q11-. *p < 0.05

Regional facial measurements are shown in Fig. 4A and Supplemental Table 2. There were main effects of diagnostic group for marginal reflex distance 1 [F(2,284) = 29.82, *p* = 1.7 5 × 10^-12^], marginal reflex distance 2 [F(2,284) = 14.10, *p* = 1.46 × 10^-6^], and the philtrum [F(2,284) = 30.80, *p* = 7.79 × 10^-13^], but not brow height (Fig. 3). Marginal reflex distance 1 in 22q11DS was shorter as compared to PS (p_FDR_=0.003) and TD (p_FDR_<0.001); PS had shorter marginal reflex distance 1 than TD (p_FDR_=0.003). Marginal reflex distance 2 in 22q11DS was shorter than PS (p_FDR_=0.003) and TD (p_FDR_<0.001); PS and TD did not differ (p_FDR_=0.28). The philtrum in 22q11DS was shorter than PS (p_FDR_<0.001) and TD (p_FDR_<0.001); PS and TD did not differ (p_FDR_=0.13).

To determine if regional facial features related to psychopathology within 22q11DS, Emotrics measurements were compared between 22q11DS + and 22q11DS- patients. Marginal reflex distance 1 [F(1,136) = 5.74, *p* = 1.79 × 10^-2^] and marginal reflex distance 2 [F(1,136) = 4.69, *p* = 3.20 × 10^-2^] were shorter in 22q11DS + as compared to 22q11DS- (Fig. 4B). Brow height and the philtrum did not differ between 22q11DS PS + and 22q11DS-. In addition, SZ [F(1,124) = 7.73, *p* = 6.27 × 10^-3^] and CR [F(1,103) = 8.59, *p* = 4.15 × 10^-3^] had shorter marginal reflex distance 1 as compared to TD, but did not differ on any other measures. No regional measurements differed between SZ and CR. Given differing distribution of race by diagnosis noted above additional analyses for F2G and Emotrics that are limited by race are presented in the Supplemental Results (Supplemental Tables 1 and Supplemental Figs. 1–2). In general, the same pattern of result remains when analyzed with race. Associations between F2G PC scores and regional facial measures were weak, suggesting these two approaches are measuring unique aspects of facial morphology in the present sample. (Supplemental Table 2).

### Predicting PS clinical status from F2G and Emotrics measurements

**Fig. 5:**
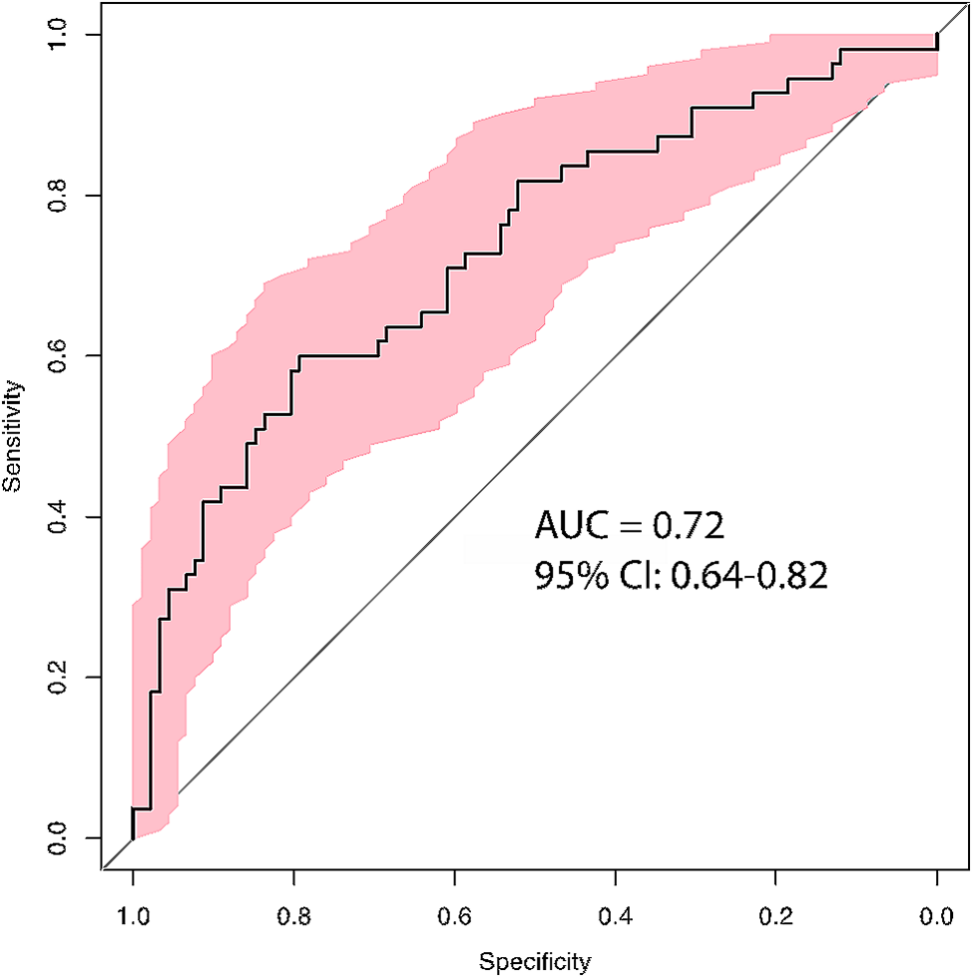
A parsimonious logistic model including PC2 and marginal reflex distance 1 was moderately effective at identifying PS from TD based upon these two features of facial dysmorphology

F2G PC scores and Emotrics measurements were used in ROC analyses to determine if any individual or combination of measurements are helpful in diagnostic classification of psychosis status. A parsimonious logistic model was built using a standardized approach [46](See Supplemental Methods). Variables of interest included demographics, PC scores and Emotrics measures. First, univariate comparisons for each variable of interest were compared between TD and PS; only those passing a threshold p-value of 0.25, per the published recommendations for forward regression [47, 48], passed to a multivariate model. The full multivariate model included all variables except, sex, BH and PC4. Results of the multivariate analyses indicated that only marginal reflex distance 1 and PC2 significantly predicted group status. Likelihood ratio test indicated similar fit for both the full multivariate model and reduced model (*p* = 0.19) containing only marginal reflex distance 1 and PC2. Interactions in the reduced model were not significant (*p* = 0.17) and model fit was good (Hosmer and Lemeshow goodness of fit test: *p* = 0.07). A leave-one cross-validated ROC of the final logistic model (marginal reflex distance 1 + PC2) had an AUC of 0.72 (95% CI: 0.64–0.82) with sensitivity of 60% and specificity of 79% (Fig. 5). Overall, results indicate that shorter marginal reflex distance 1 and higher PC2 scores were more indicative of PS status.

## Discussion

Overall, our findings indicate that computer-vision analysis of 2D photographs can identify facial dysmorphology patterns that are unique to 22q11DS and predictive of psychosis spectrum features in idiopathic psychosis as well. The present findings indicate that computer-vision detection of specific syndromic facial features of 22q11DS is feasible by showing high accuracy for patients with a laboratory confirmed 22q11.2 deletion. They were expectedly less accurate in distinguishing non-deleted PS and TD. Notably, PCA-derived factor scores indicated syndromic facial dysmorphology differences between 22q11DS with and without psychosis features that were comparable to those found between non-deleted PS and TD. Moreover, regional measures of midline facial features were most affected in 22q11DS, while PS individuals showed an intermediate pattern as compared to TD. Specifically, measures of the eye and mouth/nose were the most dysmorphic in 22q11DS and PS. When combined whole face syndromic factor scores and regional measures of dysmorphology were predictive of idiopathic PS group status. Taken together, the present results reaffirm and extend previous work indicating that craniofacial dysmorphology is present in and predictive of psychosis spectrum illness by replicating this effect in both 22q11DS and idiopathic PS.

Craniofacial dysmorphology in 22q11DS is present in most individuals but tends to be relatively mild and variable in appearance [1, 49]. In general, clinically ascertained craniofacial features include auricular and nasal abnormalities, ocular differences including hypertelorism and “hooded eyelids”, cleft lip/palate, asymmetric crying facies, and craniosynostosis [1]. While we did not assess these features clinically, F2G analysis indicated, on average, that 2D photographs of participants with 22q11DS aligned well with the average 22q11DS facial profile. First, we confirmed that almost all 22q11.2 deletion syndrome patients matched to the F2G 22q template (99%), but not all to the same degree (average 22q11DS Gestalt score = 0.52 +/- 0.29). Hence, there is a range of facial features associated with 22q11DS and no one specific feature is universal or essential. As such many of these faces match better on other syndromes. By incorporating other potential matches in facial features we have taken advantage of all of the data provided by F2G algorithm to explain more variance in facial features in 22q11DS and likely improve prediction of 22q-like features in PS or TD individuals. This underscores the importance of using computer-vision approaches to detect subtle abnormalities and the potential grouping of these abnormalities. This pattern of results suggests that our 22q11DS sample is indeed representative of facial dysmorphology typically found in the condition.

To better capture variability in syndromic facial features, a PCA analysis of all F2G-detected syndromes was used to generate factor scores that could be used to compare 22q11DS to non-deleted PS and TD. 22q11DS differed from PS and TD in two PCA-derived factor scores. 22q11DS had higher PC1 factor scores, which appear to be influenced by a strong positive loading for 22q11DS, and lower PC4 factor scores, which were anchored by a positive loading for Rett Syndrome and moderate negative loading for Silver-Russell Syndrome, which has significant facial dysmorphology including a triangular face, prominent forehead, a small jaw, and downturned corners of the mouth. Notably, previous studies of 2D and 3D facial surfaces in 22q11DS report dysmorphology patterns that are similar, including retrusive lower part and prominent upper parts of the face as compared to TD [49, 50].

Regional measures of most but not all midline facial features were smaller in 22q11DS. Measurements of the eye— marginal reflex distance 1 and 2—and of the nose—philtrum—were significantly smaller in 22q11DS as compared to both PS and TD. These regional difference add specificity to 22q11DS-related facial dysmorphology and align with previous surface-based measurements of the face that indicate abnormalities of the eye, including short down slanting palpebral fissures [50, 51] and hooded upper and lower eyelids [1], as well as upward and forward displacement of the nose, increased nasal length, narrowing of the nasal root with abnormalities of the nasal tip, narrowing of the nasal base [50], and shorter philtrum length [51]. Altogether, the present results indicate that while facial dysmorphology in 22q11DS is mild and variable, common features of facial dysmorphology are detectable using computer-vision analysis and regional abnormalities of the eye and nose may be of relevance in 22q11DS.

Importantly, F2G PCA factor scores and regional facial measures were associated with psychosis spectrum status in both 22q11DS + and non-deleted PS. 22q11DS + showed elevated PC2 scores as compared to 22q11DS PS-, and these scores did not differ from non-deleted PS. Higher PC2 scores were also found in PS as compared to TD. This pattern suggests that the variability captured by PC2 may be sensitive to psychosis. Marginal reflex distance 1 and 2 measures were shorter in 22q11DS + than 22q11DS-, while only marginal reflex distance 1 was shorter in PS as compared to TD. Moreover, the combination of PC2 and marginal reflex distance 1 were significantly predictive of PS status in a parsimoniously built logistic model. As such, the present findings agree with recent evaluations of MPAs in psychiatric conditions, including psychosis. Dysmorphic features, as rated using the Waldrop Physical Anomaly Scale [52, 53], are more common in psychosis and bipolar disorder, suggesting similar etiopathogenic origins [31]. Previous literature also indicates a higher prevalence of craniofacial dysmorphology in SZ [30, 52, 54–56]. Indeed, craniofacial dysmorphology is associated with a two-fold increase in psychosis [30]. However, other studies note that while craniofacial dysmorphology is elevated in psychosis, these MPAs appear at a similar rate as those found in other body parts [57]. Nonetheless, craniofacial dysmorphology in 22q11DS PS + and non-deleted PS likely results from a common developmental mechanism—disrupted migration of neural crest cells [58, 59]. Abnormalities in neural crest cells, which are located proximal to the neural tubes, have been implicated in craniofacial dysmorphology [54, 56]. Notably, much of the pathology related to congenital physical dysmorphology in 22q11DS can be attributed to complications in morphogenesis and subsequent abnormal function of pharyngeal arch system derivatives, including the craniofacial structures [1]. These structures receive contributions from all three germ layers of the embryo—the endoderm, mesoderm, and ectoderm—together with neural crest cells derived from the closing neural tube. Thus, it appears that subtle facial dysmorphology may be a marker of psychosis that can be linked to critical embryological time periods, which may provide promising entry points for detecting neurodevelopmental neuropathology associated with 22q11DS and psychosis, predicting risk for psychiatric symptoms and eventually helping inform precision medicine approaches to treatment.

## Limitations

Our use of a large, well-characterized sample provides a valuable perspective for determining the utility of computerized analysis of facial dysmorphology in 22q11DS and idiopathic psychosis. However, this study has limitations that should be carefully considered.

### Methodological limitations

Collecting 2D photos is simple and does not require specialized and expensive equipment, but 2D images are limited to measuring distance along the x and y axis of a given face. The use of 3D images would likely better inform us about the underlying biology as critical information about depth of the facial structures would be captured. In fact, 3D facial analysis has shown better accuracy than 2D in discriminating syndromes with facial dysmorphology [60]. Notably, advancements in mobile technology and machine learning are likely to reduce costs and increase accessibility of 3D facial capture soon. Also, we did not clinically assess facial dysmorphology using standardized measures thus direct comparison to some previous literature is difficult. However, we believe that the general patterns of facial dysmorphology are similar and that a non-biased computerized approach is an improvement upon qualitative inspection only.

### Clinical heterogeneity

It is likely that clinical heterogeneity, especially within the PS and 22q111DS groups, could affect our results. Here, we considered all of those on the psychosis spectrum (high-risk to DSM schizophrenia (SZ)) to be PS. We acknowledge that, clinically, all of the individuals are unlikely to have identical clinical feature but believe that they are on the same clinical spectrum. Given this limitation we also provide exploratory analyses of facial scores for more distinct subgroup, including SZ patients and clinical risk (CR) patients. These analyses, while completed in fewer subjects show that for some, but not all facial features, CR and SZ show similar patterns (e.g., marginal reflex distance 1 differences) and are representative of the larger PS grouping. In addition, PS individuals were enrolled form the community, but not directly tested for 22q11DS. Thus, it is possible there could be undetected 22q11DS within this sample, but we would expect this rate to be quite low given the thorough clinical assessments performed and the presence of 22q11DS in idiopathic PS is low [33]. Moreover, it is possible that there is bias in the recruitment of 22q11DS and PS willing to participate in this study. We note that the barriers to participation for this study were low (only a picture needed to be taken) but it remains possible that patients with more significant illness, and as such differing facial dysmorphology, may be underrepresented in both of these groups. It is also possible that duration of illness or other clinical factors may affect facial structure. Finally, our samples are relatively small for prediction analyses and future studies should significantly increase sample size to improve prediction. As a result, generalizing to clinical settings should be done with caution and efforts should be made to engage all patients presenting for 22q11DS or PS research or clinical services.

### Heterogeneity of participant characteristics

The present study included a wide age range of participants and diverse ethnicities; statistical approaches were used to attempt to mitigate the influence of these factors and race-specific sensitivity analyses were completed to corroborate the overall results. This difference in age was expected and is simply a reflection of that natural course of 22q11DS and PS. Patients with 22q11.2 deletion syndrome can be detected at birth since there is a known genetic deletion. Thus, these individuals are identified very early in life. PS individuals however are not typically identified until mid-to-late adolescence as psychosis is not typically identified before the age of 12. Moreover, including childhood psychosis may have further increased clinical heterogeneity since it is often the result of other psychiatric conditions (e.g. depression, ADHD, autism spectrum disorders) or is secondary to a variety of medical conditions [61]. While it is possible that age or ethnicity can affect facial measurements, a recent analysis of patients with 22q11DS found a similar pattern of MPAs in a Mexican cohort [51] suggesting similar effect sizes in 22q11DS MPAs across certain ethnicities. Thus, future work measuring longitudinal change in facial structure across age and ethnicities will better elucidate ontogenetic processes that may lead to dysmorphogenesis in 22q11DS or PS. In addition, Given the paucity of screening tools that are specific to differentiating risk for psychosis in both 22q11DS and idiopathic psychosis, we believe that this study provides results that may improve detection of those who may be at greater risk for psychosis.

In conclusion, our results suggest that computer-vision analysis of 2D facial photographs indicates overlap in facial dysmorphogenesis between patients with 22q11DS and PS. Future work linking measures of facial dysmorphology, preferably using 3D imagery, to clinical and neurobiological phenotypes will help identify features most directly linked to psychosis and psychosis risk. Finally, to the extent that these developmental markers are evident before sub-psychotic symptoms are evident, they may also allow more reliable identification of psychosis risk.

### Electronic supplementary material

Below is the link to the electronic supplementary material.

Supplementary Material 1

## Data Availability

The datasets generated and/or analyzed during the current study are not publicly available due to the fact that the primary data are facial pictures of participants, which by definition cannot be deidentified. As such, primary data cannot be made publicly available. Please contact Dr. David Roalf with questions and considerations for data sharing upon reasonable requests.
