# Supplementary material for: Computer-vision analysis of craniofacial dysmorphology in 22q11.2 deletion syndrome and psychosis spectrum disorders
Source: J Neurodev Disord. 2024 Jun 25;16:35. doi: 10.1186/s11689-024-09547-8 (PMC11201300; doi:10.1186/s11689-024-09547-8)
Supplement: Supplementary file 1 — Supplementary Material 1 [file 11689_2024_9547_MOESM1_ESM.docx]

**Supplemental Material in support of**

**Computer-vision analysis of craniofacial dysmorphology in 22q11.2 deletion syndrome and psychosis spectrum disorder.**

David R Roalf^1, 2^, Donna M. McDonald-McGinn^3^, Joelle Jee^1, 2^, Mckenna Krall^1, 2^, T. Blaine Crowley^3^, Paul J. Moberg^1^, Christian Kohler^1^, Monica E. Calkins^1, 2^, Andrew J.D. Crow^1^, Nicole Fleischer^4^, R. Sean Gallagher^1, 2^, Virgilio Gozenbach^5^, Kelly Clark^5^, Ruben C. Gur^1, 2^, Emily McClellan^1, 2^, Daniel E. McGinn^3^, Arianna Mordy^1^, Kosha Ruparel^1, 2^, Bruce I. Turetsky^1, 2^, Russell T. Shinohara^5,6^, Lauren White^1, 2^, Elaine Zackai^3^, Raquel E. Gur^1, 2^

^1^Brain Behavior Laboratory, Department of Psychiatry, Perelman School of Medicine, University of Pennsylvania, Philadelphia, Pennsylvania, USA

^2^Lifespan Brain Institute, Department of Child and Adolescent Psychiatry and Behavioral Sciences, Children’s Hospital of Philadelphia, USA

^3^22q and You Center at the Children’s Hospital of Philadelphia

^4^FNDA, Boston MA, USA

^5^Penn Statistics in Imaging and Visualization Endeavor (PennSIVE), Perelman School of Medicine, University of Pennsylvania, Philadelphia, PA, USA

^6^Center for Biomedical Image Computing & Analytics (CBICA), Perelman School of Medicine, University of Pennsylvania, Philadelphia, PA, USA

*Correspondence: David R. Roalf, Ph.D., Neuropsychiatry Section, Department of Psychiatry, 5^th^ Floor, Richards Building, 3700 Hamilton Walk, Philadelphia, PA, 19104. Telephone: (215) 662-4678. Fax: (215) 662-7903. E-mail: [roalf@pennmedicine.upenn.edu](mailto:roalf@pennmedicine.upenn.edu)

Statistical code and data available here: <https://github.com/PennBBL/craniofacial/tree/main>

**Supplemental Methods & Results**

**Principal Component Analysis:**

Given the large number of features (F2G Gestalt scores) returned by F2G the goal for performing PCA was to (1) dimensionality of analysis while capturing most of the variance in the original set of variables; (2) capture common variance across Gestalt scores, (3) use the reduced variable set (i.e. principal components) for prediction.

To ensure that variation associated with 22q11DS was captured we performed PCA original set of Gestalt scores from F2G. The Gestalt scores were not normalized, as they already ranged from 0 to 1, but more importantly normalization was not performed in order to preserve the magnitude of the relative variance between Gestalt scores. Lastly, based on visual scree plot analysis it was determined that the first 4 principal components should be retained (Supplemental Figure 6). We used PCA for data reduction as opposed to factor analysis, where rotations are commonly applied to produce orthogonal or oblique factors with the aim of measuring and describing distinct constructs by relating them to the original values. In our analysis, first we wanted to test each feature (PC) for its predictive value and then relate the feature to the original values by examining the loadings. Therefore, no rotation was applied to maintain the maximum variance rankings—1^st^ component will have the most variance, 2^nd^ has max variance after it, and so on…—and iteratively evaluate each component for prediction. While rotation can aid in interpretability in factor analysis by simplifying the structure of factors, the primary goal of PCA in our study was dimensionality reduction and variance preservation, which does not necessitate rotation. Furthermore, rotation would partition variance more evenly between the four PCs, however, we are not equally interested in all instead our goal is to interpret the components that are potentially predictive of psychosis.

We excluded Gestalt scores that were “0” **across all individuals in the 22q11DS sample**. How could this happen? 1) The Top 30 syndrome hits were selected by F2G for each individual, however, in some instances hits were present in PS or TD but never present in 22q11DS. While these syndromes could be of interest they were not of interest in current analysis since our question was focused on whether syndromes **present** in 22q11DS are predictive of psychosis in the general population. Ultimately, there were 227 syndromes that met this criteria.

To apply the PCA model to out-of-sample groups (PS and TD), we used the loadings derived from the 22q11DS group to calculate the scores as weighted sums of the original Gestalt scores for each principal component (PC1, PC2, PC3 and PC4). This approach ensures that we maintain the independence of the validation process while leveraging the dimensional reduction and feature extraction performed above.

**Investigation potential effects of race on facial measurements:**

The known differences in facial appearance with ethnicity across human populations may challenge the capability of statistical techniques to control for these differences. Given differing distribution of ethnicities by diagnosis noted in the main text additional analyses were completed for F2G and Emotrics measures that restricted to within and across ethnicities.

First, data was limited to the two racial groups with the largest sample size. The sample included 14 22q11DS, 29 PS and 37 TD Black/African Americans and 129 22q11DS, 20 PS and 54 TD Caucasians. F2G Gestalt scores for 22q11.2 deletion syndrome (Table 1) did not differ by race (p=0.07) nor was there an interaction between diagnostic group and race. As can be seen in Supplemental Figure 1 the pattern of results for this F2G Gestalt score is higher in 22q11DS individuals and similarly low in PS and TD. This patterns appears to be independent of race

**Supplemental Table 1**

|  | **Black/African American** | **White** | **All participants** |
| --- | --- | --- | --- |
| **22q11DS** | 0.62 (0.29) | 0.51 (0.29) | 0.51 (0.29) |
| **PS** | 0.17 (0.09) | 0.08 (0.11) | 0.13 (0.13) |
| **TD** | 0.18 (0.11) | 0.10 (0.11) | 0.14 (0.12) |

**Supplemental Figure 1. F2G 22q11.2 Gestalt scores by race.**

Similar analyses were completed for Emotrics measurements. There was a main effect for race in Brow Height F(1, 268)=20.99, p=7.08x10^-6^ and MRD2 F(1, 268)=14.08, p=2.14x10^-4^, but not for MRD1 or Philtrum. Only MRD2 showed a group x race interaction where Caucasian, but not Black/African Americans, 22q11DS individuals showed lower MRD2 as compared to PS or TD (Supplemental Figure 2).

**Supplemental Figure 2. Emotrics outcome measure by race.**

**Non-AD deletion facial dysmorphology**

It is also possible that deletion length could affect facial developmental patterns. As such facial measurements were compared between typical 22q11DS (A-D deletion) and those with other deletions (non-A-D). There were six non-A-D deletion 22q11DS individuals included in the overall sample. Five of six were detected by F2G as having 22q11DS but with a lower average Gestalt score (0.14+/-0.06) than AD (0.53+/-0.28). The highest Gestalt score was for Neurofibromatosis, Type 1 (0.21). Emotrics outcome measures were Brow height = 21.21 (4.84), MRD1 = 1.88 (0.43), MRD2 = 3.48 (1.54), and Philtrum = 13.41 (5.36), none of which differed from Emotrics values in the AD deleted group: Brow height = 20.35 (4.50), MRD1 = 2.26 (1.16), MRD2 = 3.97 (1.75), and Philtrum = 13.78 (4.49). Sensitivity analysis removing the six non-AD 22q11DS participants does not alter the results reported in the main text.

**Building a parsimonious logistic model for prediction**

F2G PC scores and Emotrics measurements were used in ROC analyses to determine if any individual or combination of measurements are helpful in diagnostic classification of psychosis status. As noted in the main text a parsimonious logistic model was built using a standardized published approach (Zhang, 2016) The steps and results are detailed below

**Mass univariate analysis.** The unadjusted associations between all predictor variables and the outcome if interest—PS or TD—were modeled. Predictors of interest included age, sex, race, BH, MRD1, MRD2, PHL, PC1, PC2, PC3 and PC4. Univariate significance values are provided for each predictor in the table below.

| Predictor | p-value |
| --- | --- |
| *age* | *0.002* |
| sex | 0.262 |
| *race* | *0.123* |
| BH | 0.336 |
| *MRD1* | *0.000* |
| *MRD2* | *0.237* |
| *PHL* | *0.002* |
| *PC1* | *0.239* |
| *PC2* | *0.001* |
| *PC3* | *0.156* |
| PC4 | 0.827 |

Predictors passing a liberal threshold p-value of 0.25 (italics) passed to the next step. This p-value was selected based on published recommendations (Bendal and Afifi, 1977; Mickey and Greenland, 1989; Zhang, 2016)

**Multivariate model comparison.** All predictors that passed univariate analysis were entered into one multivariate model. Any predictor that did not contribute to the model significantly (p<0.05) is eliminated. A reduced model containing only those significant predictors is then computed. The two models are then compared using partial likelihood ratio test to ensure that the reduced model fit is equivalent to the full model.

| Full Model | |  | Reduced Model | |
| --- | --- | --- | --- | --- |
| Predictor | p-value |  | Predictor | p-value |
| age | 0.129 |  | MRD1 | <0.000 |
| race | 0.150 |  | PC2 | <0.000 |
| MRD1 | 0.001 |  |  |  |
| MRD2 | 0.376 |  |  |  |
| PHL | 0.139 |  |  |  |
| PC1 | 0.968 |  |  |  |
| PC2 | 0.036 |  |  |  |
| PC3 | 0.103 |  |  |  |
|  |  |  |  |  |
| Model AIC | 168.190 |  | Model AIC | 164.030 |
|  |  |  |  |  |
|  | Log Likelihood Ratio Test: X(-7)=9.83, p=0.19 | | | |

Only MRD1 and PC2 were included in the final multivariate model. Model AICs were similar and the log likelihood ratio test comparing model fits was not significantly different. Notably, the variable selected were not impacted by race effects.

As such, the reduce model was accepted.

**Interactions between predictors were tested**. The new model containing an interaction was then compared to the model without the interaction using partial likelihood ratio test.

| Model-no interaction | |  | Model - interaction | |
| --- | --- | --- | --- | --- |
| Predictor | p-value |  | Predictor | p-value |
| MRD1 | 0.000 |  | MRD1 | 0.001 |
| PC2 | 0.000 |  | PC2 | 0.749 |
|  |  |  | MRD1*PC2 | 0.187 |
|  |  |  |  |  |
| Model AIC | 164.030 |  | Model AIC | 164.210 |
|  |  |  |  |  |
|  | LogLikelihood RatioTest: X(1)=1.18, p=0.17 | | | |

Inclusion of the interaction term did not improve model fit. Model AICs were similar and the log likelihood ratio test comparing model fits was not significantly different. As such, the interaction term was no included in the model.

**Goodness of fit.** Last, Hosmer-Lemeshow goodness of fit test was used to assess the fit of the final model. The p-value of the Hosmer-Lemeshow test was not significant (p=0.08) indicating that there is no significant difference between observed and predictive values.

**Supplemental Figure 3.** Average Face2Gene Gestalt scores for 22q11DS participants. 99% of 22q11DS photographs matched to the F2G 22q11DS gestalt. Other syndromes that matched F2G Gestalts in at least 50% of 22q11DS participants are displayed.

**Supplemental Figure 4.** Average Face2Gene Gestalt scores for non-deleted PS participants. Syndromes that matched F2G Gestalts in at least 50% of PS participants are displayed.

**Supplemental Figure 5.** Average Face2Gene Gestalt scores for non-deleted TD participants. Syndromes that matched F2G Gestalts in at least 50% of TD participants are displayed.

**Supplemental Figure 6.** Scree plot for the principal component analysis on F2G syndromes pres. The first four principal components were selected for further analysis.

**Supplemental Figure 7.** F2G syndromic contributions for each principal component score.

**Supplemental Figure 8.** PCA pattern plots. Pattern plots are shown for consecutive components. Plots include data for 22q11DS (orange), PS (pink) and TD (blue). Triangles represent the mean PC score for a given group.

**Supplemental Table 2.** Average measurement values for Brow Height, Marginal Reflex Distances 1 and 2, and Philtrum. Measurements were made using Emotrics.

**Supplemental References**

Bendal, R., Afifi, A., 1977. Comparison of stopping rules in forward regression. J Am Stat Assoc 72 (357), 46-53.

Mickey, R.M., Greenland, S., 1989. The impact of confounder selection criteria on effect estimation. American Journal of Epidemiology 129 (1), 125-137.

Zhang, Z., 2016. Model building strategy for logistic regression: purposeful selection. Annals of translational medicine 4 (6).
